# Angle grinder injuries in orthopedics: A case series and review of the literature

**DOI:** 10.1016/j.tcr.2021.100413

**Published:** 2021-02-10

**Authors:** M.G. Curran, K. Clesham, S. Irwin, A. Hughes, M. Kennedy

**Affiliations:** a Univesirty of Limerick School of Medicine, University of Limerick, Dooradoyle, Co. Limerick, Ireland; b Department of Orthopedics, Midland Regional Hospital Tullamore, Co. Offaly, Ireland

**Keywords:** Angle grinder, Work related injury, Trauma, Orthopedics

## Abstract

Angle grinders are amongst the most dangerous tools used in industry and agriculture. Over 5000 documented injuries are related to their use each year which are commonly triggered by a shattering of the abrasive wheel. These injuries are often accompanied by suboptimal health and safety standards. The authors of this paper present three separate cases of accidental injuries presenting to our institution over a short time period. The authors main aim is to raise awareness surrounding the associated dangers of using such tools. A brief economic analysis also illustrates the significant costs involved in treating such preventable injuries.

## Background

Angle grinders are powerful hand tools widely used in a variety of work environments. Their high-speed revolving disc can cut, grind and polish metal, concrete or other hard surfaces [1]. ‘The Royal Society for the Prevention of Accidents’ Accident Surveillance Systems data ranked angle grinders as the third most dangerous tools, with 5400 injuries recorded annually [2]. The vast majority of these injuries are caused by a shattering of the abrasive wheel [3]. Several case studies available in the literature suggest a propensity towards head or eye injuries [3] [4], however upper limb injuries are also common [1].

The authors of this study present three separate cases of accidental injury caused by angle grinders presenting to our institution over a short period of time. The cases describe a series of orthopaedic injuries highlighting the musculoskeletal consequences of improper use of angle grinders and the significant morbidity they bear. The authors also examine the significant amount of resources used in the treatment of such injuries and aim to raise awareness surrounding the dangers associated with the improper use of such tools.

## Case 1

**Fig. 1:**
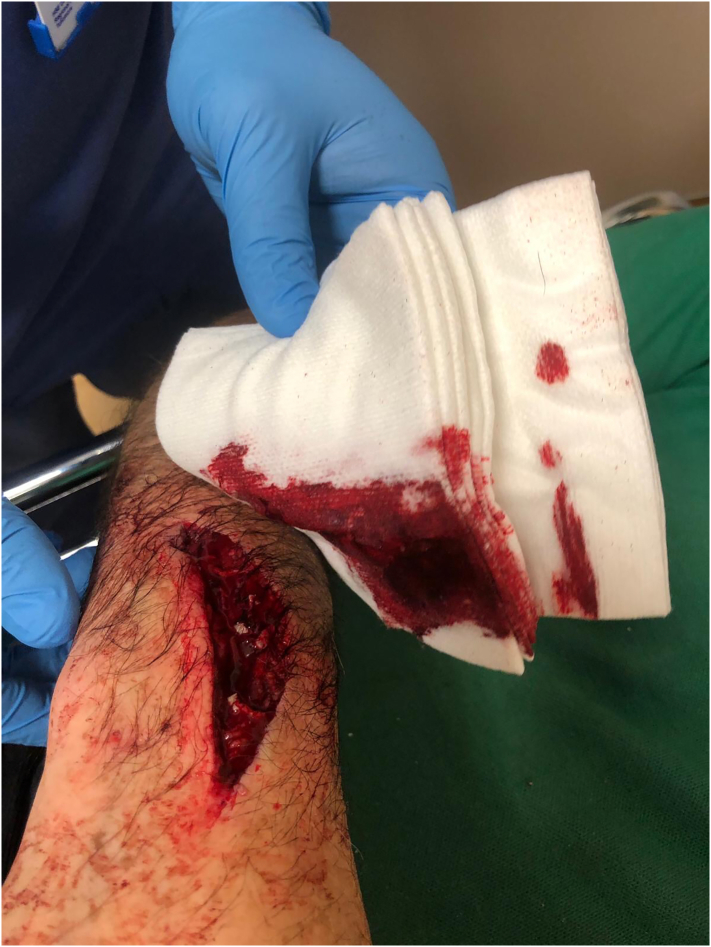
Right forearm laceration showing extensive muscle belly damage.

Case 1 describes the injury of a 58 year-old left-handed male. He was using an angle grinder at home to manufacture a frame when he lost his grip and sustained a direct laceration to the right forearm (Fig. 1). He underwent a washout and debridement of the wound on the same day of his admission. Surgical exploration demonstrated significant damage to the brachioradialis muscle belly. Intact neurovascular status was confirmed post-operatively demonstrating nearby anatomical structures such as the superficial branch of the radial nerve and the radial artery remained uninjured.

## Case 2

Case 2 describes the injury of a 49 year-old left-handed male. He sustained a deep laceration to his right foot while cutting steel with an angle grinder. He described a definite bouncing incident prior to blade failure. This resulted in loss of control and eventual injury. Of note, he was not wearing protective footwear at the time of the incident.

**Fig. 2:**
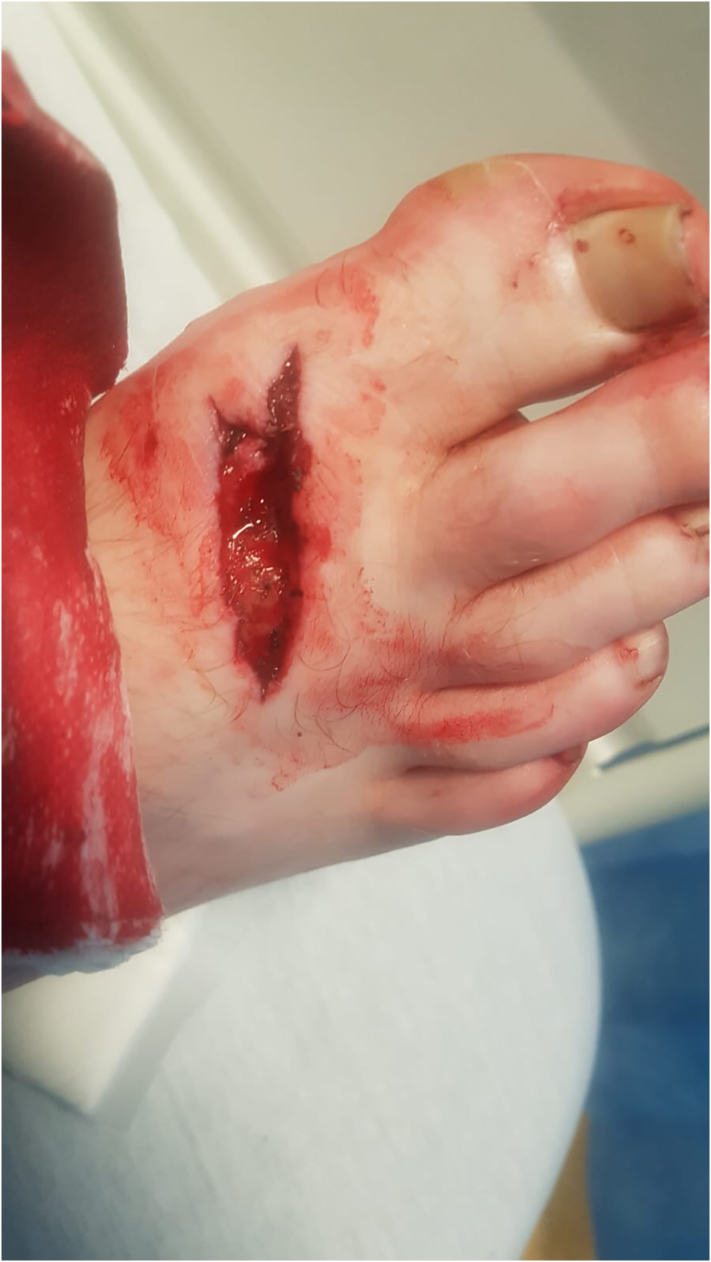
Deep laceration to the dorsum of the right foot.

Fig. 2 shows a 6cm, deep laceration to the dorsum of his right foot. On examination he was unable to dorsiflex his hallux against resistance but was otherwise neurovascularly intact. A complete laceration of the extensor hallucis longus was identified on surgical exploration. A small bone fragment was also excised during washout. The extensor hallucis longus tendon was repaired using the modified Krackow technique. The patient was placed in a walking boot for six weeks and encouraged to heel weight-bear.

## Case 3

**Fig. 3:**
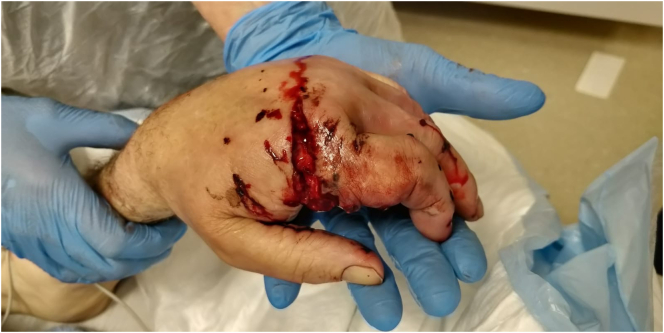
Clinical presentation of left hand injury.

**Fig. 4:**
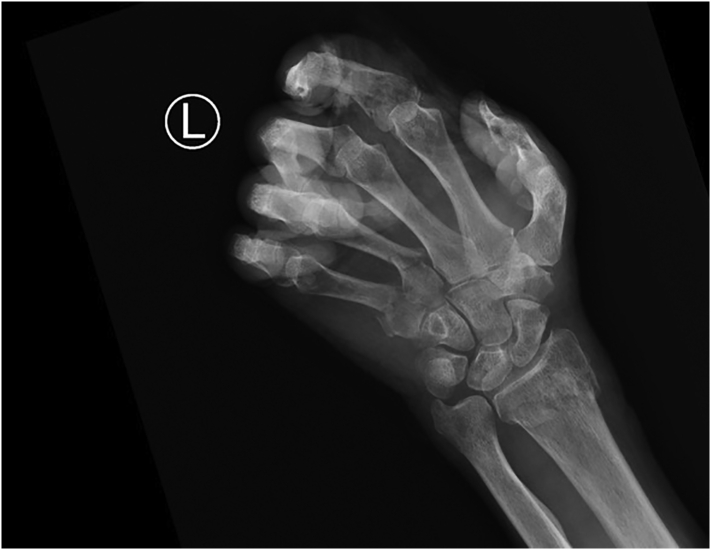
Posterior anterior radiograph of left hand showing numerous fractures.

**Fig. 5:**
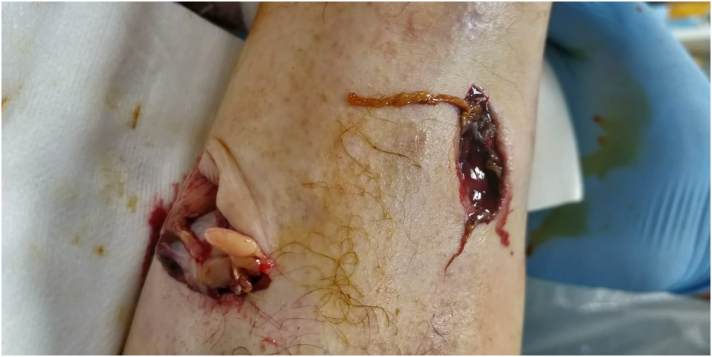
Clinical presentation of right leg.

**Fig. 6:**
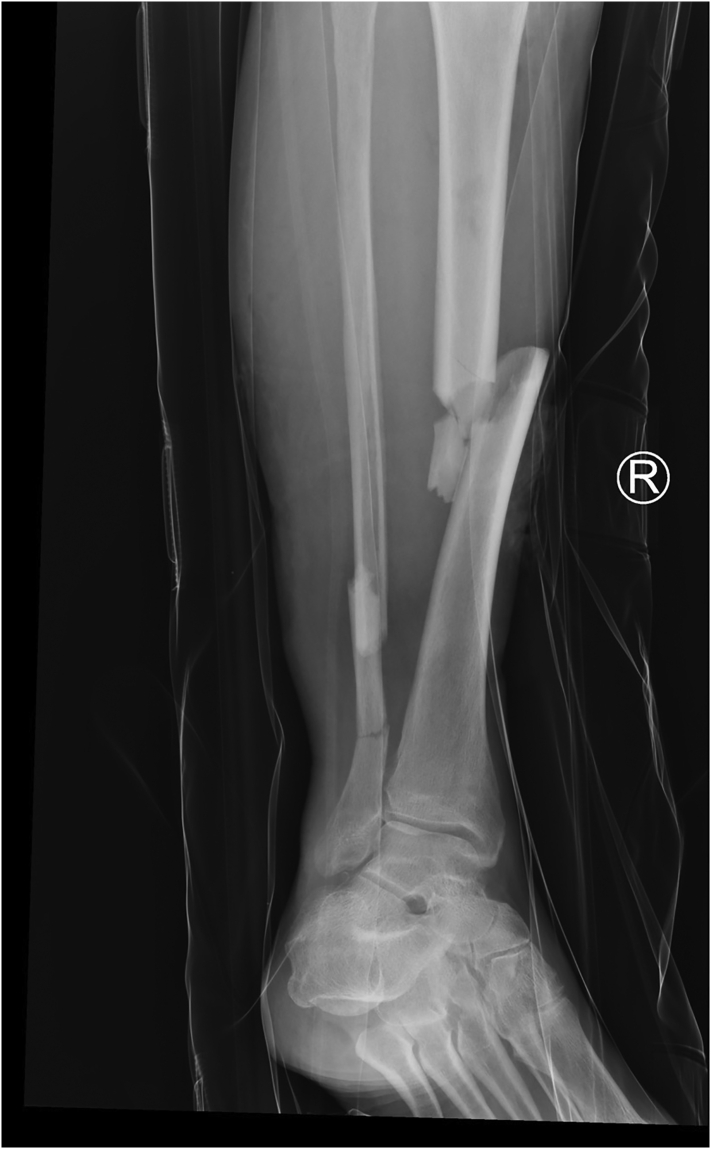
Anterior posterior radiograph of the right leg showing a comminuted midshaft fracture of the tibia with a minimally displaced comminuted fracture of the fibula.

**Fig. 7:**
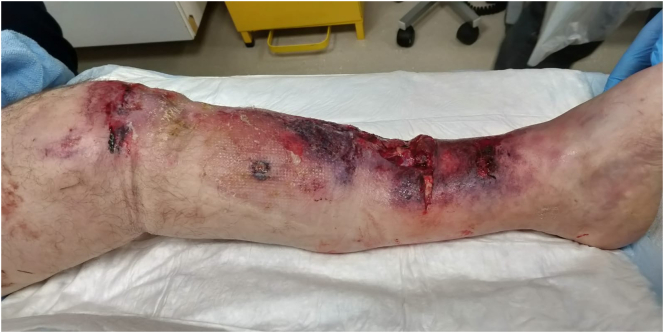
Clinical presentation of left leg.

**Fig. 8:**
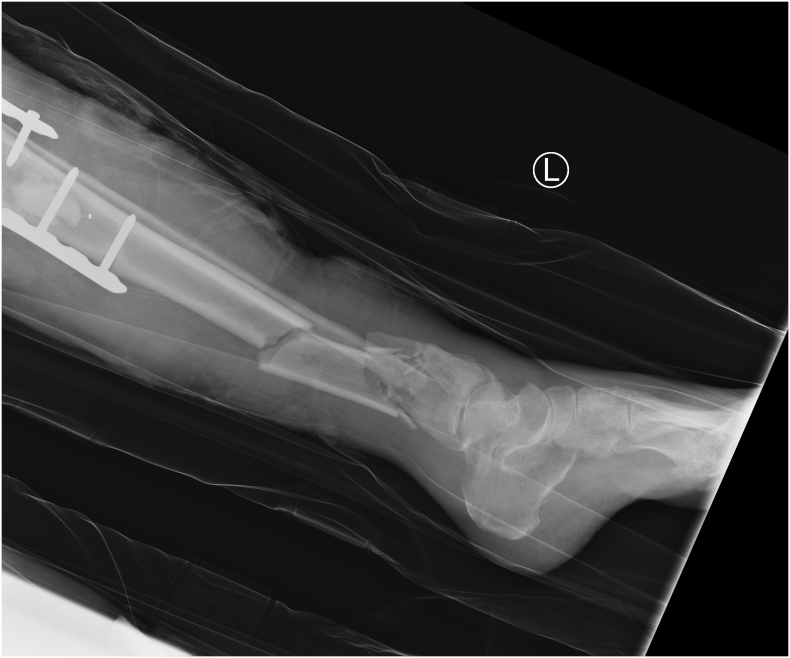
Lateral radiograph of the left leg showing a comminuted fracture of the tibia + fibula with metalwork from previous knee arthrodesis in situ.

Case 3 describes the significant injuries sustained by a 65 year-old right-handed gentleman who was using an angle grinder to make a cattle trough. He described an initial slip causing a direct injury to his left hand (Fig. 3, Fig. 4). He then reports a subsequent explosive injury following contact with a nearby fuel tank culminating in bilateral open tibia and fibula fractures. (Right leg = Fig. 5, Fig. 6; Left leg = Fig. 7, Fig. 8). He also sustained an open left index finger proximal phalanx fracture, a distal radius fracture and minimally displaced fractures to both the fourth and fifth metacarpals.

**Fig. 9:**
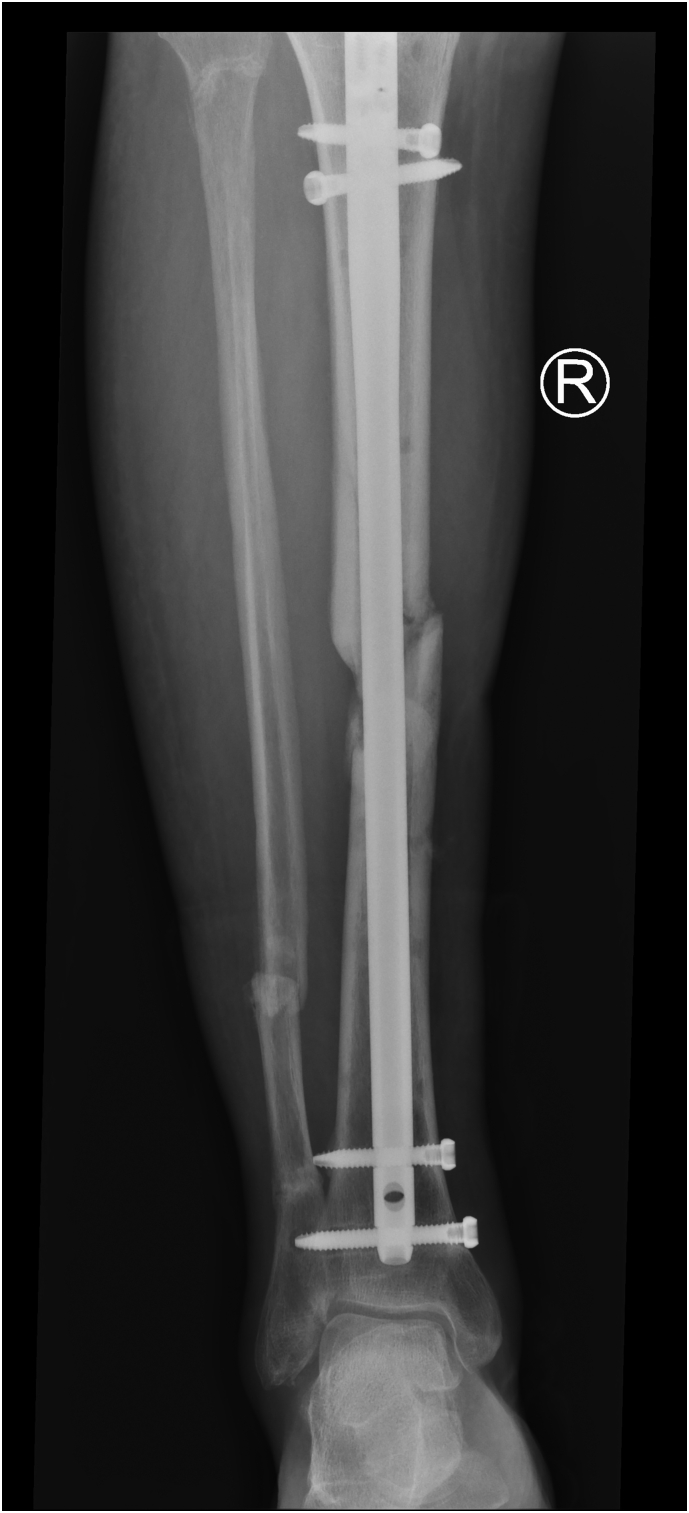
Anterior posterior radiograph of the right leg showing IM nail in situ.

He underwent left tibia/fibula external fixation, right tibia intramedullary nailing (Fig. 9) and a left hand manipulation under anaesthesia. He was later transferred to a tertiary plastic surgery unit for split-thickness skin grafting of the wounds on his left lower leg. After eight weeks the external fixator was removed and converted to open reduction internal fixation (ORIF). This subsequently required removal due to infection three months post ORIF. His recovery was further complicated by a pulmonary embolism eight months after the initial injury. This required another hospital admission and long-term anticoagulation.

In total this gentleman required five inpatient stays totalling 141 days of inpatient care. He required multiple procedures, prolonged courses of antibiotics, close monitoring of wounds and extensive rehabilitation. He underwent six procedures in four separate operations and required one outpatient appointment. Fortunately, he has been successfully discharged to a rehabilitation facility but still requires the use of a wheelchair for mobilising outside the home environment.

## Discussion

All three cases presented to our institution within a three-month period suggesting a widespread misuse of such dangerous tools in the community. Furthermore, their occurrence may indicate another influx of accidental DIY injuries previously quoted in the literature [5]. Each case posed its own level of clinical complexity and demonstrates how labour intensive treating such injuries can be, successfully reinforcing the importance of correct usage of dangerous equipment. Furthermore, with a distinct lack of prevention messages in media reports pertaining to serious agricultural based injuries [6] more awareness is critical.

The need for basic principles of safe use of such power tools should be conveyed in the community to reduce the risk of similar injuries in the future. Regular maintenance of such tools is also imperative. Users should also ensure the utilisation of the angle grinder protective guard and position themselves perpendicular to the plane of the cutting wheel in order to reduce the risk of avoidable injuries [4]. UK national guidelines [7] highlight operating precautions and guidance on personal protective equipment which is summarised in Table 1.

**Table 1:** Personal protective equipment and the advantages of their correct use.

Personal protective equipment	Advantages
Relevant eye protection	Protects eyes against flying abrasive and metallic particles
Face masks	Avoids inhalation of dust
Avoidance of loose clothing (ties/coat sleeves)	Avoids unnecessary injuries due to loose clothing being easily drawn into the wheel
Adequate head protection	Protects the operator from wheel fragments or debris
Protective gloves	Protects the operator from unintentional injury or lessens subsequent injury
Protective footwear	Protects the operator from unintentional injury or lessens subsequent injury

As alluded to previously the treatment of such injuries result in significant costs to the health service. Extrapolation of such costs can prove difficult. However, the authors of this study have included a rudimentary economic analysis of the burden posed by these injuries (Table 2). The figures quoted have been extracted from the 2019 version of Activity Based Funding released by the Health Service Executive in Ireland [8] and include surgical costings, associated average length of stay and the cost of an outpatient appointment (€129) and overnight stays (€839).

**Table 2:** Total estimated costs involved in treatment.

Pt	Surgery costs (€)	Added/subtracted costs (€)	Price per patient (€)	Total (€)
1	6074	258	6332	
2	6466	516	6982	
3	124,141–130,368	5992	130,133–136,360	
				143,447 - 149,674

## Conclusion

The three patients described in the case series portray some of the intricacies involved in providing adequate assessment and treatment for such complex injuries. Furthermore, the exceptionally high costs associated with treating their injuries demonstrates the importance of adequate education and training before using such dangerous equipment. The discussion surrounding their care also adds to the current literature base which mainly focuses on upper limb and facial injuries which are more commonly associated with angle grinders injuries.

## References

[1] Back D.L., Espag M., Hilton A., Peckham T. (2000 Jul). Angle grinder injuries. Injury..

[2] Sozbilen M.C., Dastan A.E., Gunay H., Kucuk L. (2018). A prospective study of angle grinder injuries in the hands and forearms during a one-year period. Hand Surg Rehabil..

[3] Carter L.M., Wales C.J., Varley I., Telfer M.R. (2008 Jan 23). Penetrating facial injury from angle grinder use: management and prevention. Head Face Med.

[4] Khurram S.A., Atkins S., Smith K.G., Yates J.M. (2011 Jul). A multidisciplinary approach to management of extensive facial injuries resulting from the use of an angle grinder. Inj Extra..

[5] Murphy S.M., Kieran I., Shaughnessy M.O. (2011 Sep). The trauma of a recession. Ir. J. Med. Sci..

[6] Randall J.R., Pennetta De Oliveira L., Belton K., Voaklander D. (2020 Jan). Agriculture-related Injuries: discussion in Canadian media. J Agromedicine..

[7] Health Service Executive (HSE) (2000). Safety in the use of abrasive wheels. Revised in line with the Provision and Use of Work Equipment Regulations 1998 (PUWER 98).

[8] Health Pricing Office (HPO). ABF 2019 Admitted Patient Pricing List. DRG Prices for Inpatients and Daycares 2019. HSE; 2019.

